# Oxygen Saturation in Relation to Flying Altitude. A Scoping Review Protocol

**DOI:** 10.1111/aas.70041

**Published:** 2025-04-22

**Authors:** Joachim Kvernberg, Finn Lund Henriksen, Kasper Glerup Lauridsen, Marius Rehn, Peter Martin Hansen

**Affiliations:** ^1^ Department of Cardiology Odense University Hospital Odense Denmark; ^2^ Aarhus University Hospital Aarhus Denmark; ^3^ Oslo University Hospital Prehospital Division Oslo Norway; ^4^ Norwegian Air Ambulance Foundation Oslo Norway; ^5^ Department of Anesthesiology and Intensive Care Medicine, Odense University Hospital Svendborg Svendborg Denmark; ^6^ Prehospital Research Unit, Region of South Denmark Odense Denmark; ^7^ Danish Air Ambulance Aarhus Denmark

**Keywords:** air travel, cabin pressure, hypoxia, oxygen saturation

## Abstract

**Background:**

During air travel, the decrease in air pressure leads to a decrease in oxygen partial pressure causing oxygen desaturation. Every year, several commercial aircraft need to divert and perform unscheduled landings due to hypoxic symptoms or medical emergencies. How individuals are affected depends on their physical and medical conditions, as well as the cabin pressure of the aircraft. The phenomenon is of particular concern for individuals with pre‐existing medical conditions, as it may lead to hypoxic symptoms and necessitate unscheduled landings in some cases. The investigators aim to investigate the existing literature to explore how the impact of reduced oxygen partial pressure affects the oxygen saturation as a result of a decrease in cabin pressure due to aircraft altitude, and to assess the frequency of hypoxic symptoms reported during air travel. The purpose of the scoping review is to investigate the relationship between altitude, cabin pressure, and patient oxygenation during air travel. Further, the investigators will report the frequency of the reporting of hypoxic symptoms in the studies conducted.

**Methods and Analysis:**

This scoping review will be conducted in accordance with the Cochrane Handbook and Joanna Briggs Institute Manual for Scoping Reviews. The review question will be formulated using the Population, Intervention, Comparator, Outcome, Study Design, Timeframe (PICOST) framework. Every study design is eligible, apart from reviews, meta‐analyses, comments/letters without original data, case reports with less than five cases, animal studies, and in vitro studies. The investigators will include all articles in English or Scandinavian. The investigators will base their conclusions on the findings of the review.

**Ethics and Dissemination:**

According to Danish law, scoping reviews are exempt from ethics committee approval. The investigators will publish results from the scoping review in a peer‐reviewed journal and present the results at scientific conferences.

## 1 Introduction

### 1.1 Rationale

Air travel has developed to be one of the most common ways for long journeys [1, 2, 3]. Although one of the safest transport modes, it still poses certain risks to travelers with cardiopulmonary disease [4]. Despite this, there has been debate about the paucity of evidence regarding the effect of air travel on oxygen saturation in the general population [1, 2, 3, 4, 5]. The most frequently reported medical emergencies during flights are syncope and respiratory distress [3, 4].

During air travel, the aircraft reaches cruising altitudes between 39,000 and 45,000 ft [1, 2, 3, 4]. As the altitude increases, the air density decreases [6]. As the air pressure outside the cabin decreases rapidly during the aircraft's ascent, the cabin is pressurized by pumping conditioned air from the engines' compressor stage, causing a substantively reduced decline in air pressure [2, 4, 6]. This enables passengers to breathe normally.

Depending on the type of aircraft, the cabin is pressurized to an equivalent of about 6000–8000 ft (2438 m) above sea level, resulting in a reduction in partial oxygen pressure (PaO_2_) to the equivalent of breathing 15% oxygen at sea level [1, 2, 3, 4, 6, 7]. In healthy passengers, arterial oxygen will decrease to 7.0–8.5 kilopascals (kPa), and oxygen saturation drops to SpO_2_ 85%–91% [6]. However, some specific patient categories may experience further decline in oxygen saturation [1].

The reason why the cabin is not pressurized to sea level is because it would require significantly more energy and would increase the weight of the aircraft, as the structure would need to withstand substantive pressure differences.

As the cabin pressure decreases, the PaO_2_ declines equivalently, leading to reduced oxygen supply for the passengers [1, 6]. In healthy individuals, several studies have documented varying degrees of decline in oxygen saturation [6]. However, the various effects on oxygenation in various patients undergoing air transport are poorly described. With respect to this, further research is needed to investigate the impact of air travel on this population, even though air travel guidelines have been established [1, 6].

### 1.2 Objectives

This scoping review aims to assess the existing scientific evidence on oxygenation during air travel. The investigators plan to investigate the literature reporting the extent of the decline in oxygen and hypoxia during air travel and the reported frequency of hypoxic symptoms in relation to health status concerning acute and chronic health conditions. Further, the investigators aim to describe the transferability to helicopter and airplane transport of patients [8] in the review.

## 2 Methods

The authors plan the scoping review to be in accordance with the Cochrane Handbook [9] and Joanna Briggs Institute Manual for Scoping Reviews [10]. The protocol for the scoping review adheres to the Preferred Reporting Items for Systematic Review and Meta‐analysis (PRISMA) PRISMA‐P 2015 statement for protocols [11] and the PRISMA‐P 2015: Elaboration and Explanation document [12]. The investigators developed the search strategy with the assistance of a research librarian.

Using the Covidence [13] software (Veritas Health Innovation, Melbourne, Australia), the investigators will systematically remove duplicates. Accordingly, two members of the project group will screen titles and abstracts for data extraction and subsequently perform a full‐text review. The investigators have registered the scoping review with the Open Science Framework (OSF) [14] to comply with the PRISMA‐ScR [11] guidelines for reporting scoping reviews. This scoping review protocol was registered with Open Science Framework on 27 March 2025 (registration number osf.io/4huak).

### 2.1 Eligibility Criteria

#### 2.1.1 Inclusion Criteria

Studies investigating oxygen saturation in individuals during air travel or patient transport.

#### 2.1.2 Exclusion Criteria

Reviews, meta‐analyses, comments/letters without original data, case reports with fewer than five cases, animal studies, in vitro studies, and studies with abstracts in other languages than English or Scandinavian. Studies investigate military or space aviation in relation to the effects of G‐force or weightlessness using healthy individuals.

The methodology will include formulating the review questions based on population, intervention, comparison, outcomes, study design, and timeframe (PICOST) (Table 1).

### 2.2 Information Sources

The investigators will search the following databases:

- MEDLINE (Ovid)
- Cochrane library (Wiley)
- EMBASE (Ovid)
- Scopus (Elsevier)
- WHO ICTRP
- Clinicaltrials.gov

The investigators will not impose time limits to avoid missing relevant literature. Furthermore, the investigators will do forward and backward citation searches on included studies and report the search in accordance with the PRISMA extension for searching [15].

### 2.3 Search Strategy

The search strategy will aim to identify published studies fulfilling inclusion criteria. Every study design is eligible, except for reviews, meta‐analyses, comments/letters without original data, case reports with less than five cases, animal studies, in vitro studies, and studies in other languages than English or Scandinavian.

The investigators have developed the literature search strategies in cooperation with a research librarian, using a block search comprising two search blocks (air travel and oxygen saturation); subject headings and text words. The investigators will search MEDLINE (Ovid), Cochrane library (Wiley), EMBASE (Ovid), Scopus (Elsevier), World Health Organization International Clinical Trials Registry Platform (WHO ICTRP) [16], and Clinical trial registries [17].

The investigators will also search http://www.ndltd.org, https://www.dart‐europe.org/basic‐search.php, http://www.opengrey.eu/, https://www‐base‐search‐net.ezproxy.uis.no/, and https://oatd.org for non‐indexed/grey literature. See Supporting Information: Additional material 1 for search strategy and example of a search. The searches will be reported in accordance with PRISMA extension for Searching [15].

Further, to comply with PRISMA [11] guidelines, in the event of outdated search results before publication, that is, the last search in a review is conducted more than 6–9 months before publication, the investigators will update searches by rerunning the exact same searches before the planned publication date.

As per PRISMA [11] guideline, the investigators will report as applicable:

- Database name
- Multi‐database searching
- Online resources and browsing
- Citation searching
- Contact to authors
- Other methods

No similar review has been identified through literature search or on OSF [14] and PROSPERO [18].

### 2.4 Reference Management

After the literature search, the investigators will upload every reference in the Covidence (Veritas Health Innovation, Melbourne, Australia) [13] software for proper reference management and to secure complete and transparent duplicate removal.

### 2.5 Selection Process

Two independent reviewers will screen titles and abstracts for relevance. The investigators will assess any potentially relevant study for inclusion in detail after retrieval, that is, full text review. A third reviewer will resolve any disagreements between primary reviewers at each stage of the selection process. The investigators will report the results of the search In the PRISMA flow diagram in the final scoping review. The investigators will export all relevant meta‐data from the search history to a general‐purpose open repository, such as the Zenodo [19] repository.

**TABLE 1:** PICOST statement.

Picost statement	Description (with recommended text)
Population	Individuals using any method of air travel.
Intervention	Any specific altitude or cabin pressure in simulated or non‐simulated air travel
Comparison	Any other altitude or cabin pressure incl. sea level.
Outcomes	Primary: Oxygen desaturation in relation to increasing altitude/decreased cabin pressure during air travel or simulated aircraft environment. The investigators will define significant oxygen desaturation as decrease to ≤ 90% for individuals with a normal oxygen saturation (96%–100%) prior the trial, and for individuals with an abnormal low oxygen saturation prior to trial, the investigators will define a decrease in 6% as significant measured by an oximetry or blood gas analysis. Secondary: Reported hypoxic symptoms during flight or simulated aircraft environment. Hypoxic symptoms will include dyspnea, tachycardia, restlessness, headache, confusion, and anxiety.
Study design	Randomized controlled trials (RCTs) and non‐randomized studies (non‐randomized controlled trials, cohort studies and case series with 5 ≥ cases) are eligible for inclusion.
Timeframe	All years and all languages were included if there was an English abstract. Literature search from inception until 1 April 2025.

### 2.6 Data Collection Process

Two review authors will independently extract specific, predefined details derived from the PICOST framework and key findings with relevance to the review outcomes as per research questions. A third reviewer will resolve conflicts. The investigators will enter data extraction variables into a template for an overview. Further, the investigators will address the authors of relevant studies to retrieve additional information if warranted.

### 2.7 Data Items

From the predefined data extraction instrument (Table 2), the investigators will extract data as per the PICOST items including patient outcome characteristics such as flight height and oxygen saturation, hypoxic symptoms, whether the individuals are healthy or not, and if the study was done in a normobaric or hypobaric testing environment.

**TABLE 2:** Data extraction instrument.

Study	Year	Design	Sample size	Population (healthy/sick)	Altitude range/simulated height	Oxygen supply	Oxygen saturation	Normobaric or hypobaric testing environment
Author et al.							Max: Mean: Min:	

In the event of missing or unclear information, the investigators will state that in the data extraction template. If the information is deemed critical, the investigators will contact the authors for additional information. The investigators will not make assumptions regarding the interpretation of the results of the included literature.

### 2.8 Quality Appraisal of the Included Literature

If warranted, the investigators will assess the quality of the included studies by way of an assessment instrument across two domains for internal and external validity (Table 3), using a predefined quality appraisal template, validated from known references [20, 21]. The investigators will enter the quality appraisal variables into a template for an overview (Supporting Information: Additional material 2).

**TABLE 3:** Quality appraisal template domains and items.

Domain	Items
Internal validity	Is the author employed in the field of anesthesia, intensive care or pulmonary medicine? Does the literature provide reference to where data were obtained? Does the literature provide reference to how data were obtained? Do the authors have conflicts of interest? Has an ethics committee approved the study?
External validity	Is the oximeter used to measure oxygen saturation described? Is the equipment used to simulate altitude clearly described? Is the oxygen saturation in relation to altitude or simulated altitude clearly described? Are there indications of missing data? Are other limitations discussed? Is the study design clearly explained? Are the primary and secondary outcomes clearly described?

### 2.9 Synthesis of Results

The investigators will present the extracted data in a tabular format. The format will mirror the objective and review questions of the scoping review. If applicable, the investigators will measure the reported effect for dichotomous outcomes using risk ratio (RR) with a 95% confidence interval.

Furthermore, a narrative synthesis of the included literature will support the data, including text and tables to describe the characteristics and findings of the included literature.

The investigators will conduct sub‐group analyses stratified into:

- Medical history
- Types of aircraft
- Methods of simulation

## 3 Results

The investigators will publish results from the scoping review in a peer‐reviewed journal and present the results at scientific conferences and meetings.

The investigators will present perspectives for future practice and research implications.

## 4 Discussion

The investigators aim to assess the current body of evidence regarding air travel and oxygen saturation and the possible association with hypoxic symptoms. Accordingly, the investigators will present an overview of the current knowledge in the field and discuss possible knowledge and research gaps.

The investigators will discuss the limitations of the study, such as publication and selection bias. Based on the findings on study quality as per internal and external validity, the investigators will discuss the strengths and limitations of the scoping review. Further, the investigators will discuss the generalizability and transferability of the review findings to the field of inter‐hospital transfers in aircraft without pressurized cabin.

## Author Contributions

Joachim Kvernberg conceived the study. The protocol was drafted by Joachim Kvernberg and Peter Martin Hansen and carefully revised by all authors. All authors contributed to the development of selection criteria, the risk of quality assessment strategy, and data extraction criteria. Joachim Kvernberg and Peter Martin Hansen developed the search strategy with the assistance of a research librarian. All authors read, provided feedback, and approved the final protocol manuscript.

## Conflicts of Interest

M.R. reports a consulting fee from Norwegian Air Ambulance Foundation. An ICMJE conflicts of interest disclosure form for all authors is available. P.M.H. received a general grant for Danish Air Ambulance Foundation.

## Supporting information


**Data S1.** Supporting Information.

## Data Availability

Research data are not shared. The investigators will export all relevant meta‐data from the search history to a general‐purpose open repository.
