# Supplementary material for: Oxygen Saturation in Relation to Flying Altitude. A Scoping Review Protocol
Source: Acta Anaesthesiol Scand. 2025 Apr 22;69(5):e70041. doi: 10.1111/aas.70041 (PMC12014421; doi:10.1111/aas.70041)
Supplement: Supplementary file 1 — Data S1. Supporting Information. [file AAS-69-0-s001.docx]

**Additional material**

**Additional material 1. Literature search strategy:**

Oxygen Saturation in Relation to flying Altitude.

| Block 1: | Block 2 |
| --- | --- |
| exp aviation/  flight *  air travel *  aircraft*  airplane*  aeroplane*  aerospace medicine  HEMS*  helicopter*  fixed-wing*  rotor-wing  Free text search:  (flight* OR aviation* OR air travel* OR aircraft* OR airplane* OR aerospace medicine OR HEMS* OR helicopter* OR fixed-wing* OR rotor-wing) | exp oxygen saturation/  oxygen saturation *  SpO2*  pulse oximetry *  oxygen level *  Hypoxia*  Hypoxic stress*  Oxygen deficienc*  Diffusion anoxia  Diffusion hypoxia  Free text search:  (oxygen saturation* OR SpO2* OR pulse oximetry* OR oxygen level* OR hypoxia* OR hypoxic stress* OR oxygen deficienc* OR diffusion anoxia OR diffusion hypoxia) |
|  | |

Based on Medline. Search date 17 March 2025.

| # Searches | Result | Type |
| --- | --- | --- |
| #1 exp aviation* | 41 799 | Advanced |
| #2. flight* OR aviation* OR air travel* OR aircraft* OR airplane* OR aerospace medicine OR HEMS* OR helicopter* OR fixed-wing* OR rotor-wing | 125 670 | Advanced |
| #3. 1 or 2 | 125 670 | Advanced |
| #4. Oxygen saturation*/ | 37 767 | Advanced |
| #5. oxygen saturation* OR SpO2* OR pulse oximetry* OR oxygen level* OR hypoxia* OR hypoxic stress* OR oxygen deficienc* OR diffusion anoxia OR diffusion hypoxia | 250 887 | Advanced |
| #6. 4 or 5 | 250 887 | Advanced |
| #7. 3 and 6 | 1 894 | Advanced |

**Additional material 2. Quality appraisal instrument**

|  | INTERNAL VALIDITY | | | | | EXTERNAL VALIDITY | | | | | | |
| --- | --- | --- | --- | --- | --- | --- | --- | --- | --- | --- | --- | --- |
|  | Is the author employed in the EMS organization? | Does the literature provide reference to where data were obtained? | Does the literature provide reference to how data were obtained? | Do the authors have conflicts of interest? | Has an ethics committee approved the reporting? | Is the oximeter used to measure oxygen saturation described? | Is the type of EMS dispatch system clearly described? | Is the oxygen saturation in relation to altitude or simulated altitude clearly described? | Are there indications of missing data? | Are other limitations discussed? | Is the study design clearly described? | Are the primary and secondary outcomes clearly described? |
| Paper 1 et al | ? | Y | Y | Y | ? | N | Y | Y | N | N | Y | Y |
| Paper 2 et al |  |  |  |  |  |  |  |  |  |  |  |  |
| Paper 3 et al |  |  |  |  |  |  |  |  |  |  |  |  |
| Paper 4 et al |  |  |  |  |  |  |  |  |  |  |  |  |
| Paper 5 et al |  |  |  |  |  |  |  |  |  |  |  |  |
| Paper 6 et a |  |  |  |  |  |  |  |  |  |  |  |  |
| Paper 7 et al |  |  |  |  |  |  |  |  |  |  |  |  |
| Paper 8 et al |  |  |  |  |  |  |  |  |  |  |  |  |
| Paper 9 et al |  |  |  |  |  |  |  |  |  |  |  |  |
| Paper 10 et al |  |  |  |  |  |  |  |  |  |  |  |  |
| Paper 11 et al |  |  |  |  |  |  |  |  |  |  |  |  |
| Paper 12 et al |  |  |  |  |  |  |  |  |  |  |  |  |
| Paper 13 et al |  |  |  |  |  |  |  |  |  |  |  |  |
| Paper 14 et al |  |  |  |  |  |  |  |  |  |  |  |  |
| Paper 15 et al |  |  |  |  |  |  |  |  |  |  |  |  |
| Paper 16 et al |  |  |  |  |  |  |  |  |  |  |  |  |
| Paper 17 et al |  |  |  |  |  |  |  |  |  |  |  |  |
| Paper 18 et al |  |  |  |  |  |  |  |  |  |  |  |  |
| Paper 19 et al |  |  |  |  |  |  |  |  |  |  |  |  |
| Paper 20 et al |  |  |  |  |  |  |  |  |  |  |  |  |
| Paper 21 et al |  |  |  |  |  |  |  |  |  |  |  |  |
| Paper 22 et al |  |  |  |  |  |  |  |  |  |  |  |  |
| Paper 23 et al |  |  |  |  |  |  |  |  |  |  |  |  |
| Paper 24 et al |  |  |  |  |  |  |  |  |  |  |  |  |
| Paper 25 et al |  |  |  |  |  |  |  |  |  |  |  |  |
| Paper 26 et al |  |  |  |  |  |  |  |  |  |  |  |  |
| Paper 27 et al |  |  |  |  |  |  |  |  |  |  |  |  |
| Paper 28 et al |  |  |  |  |  |  |  |  |  |  |  |  |
| Paper 29 et al |  |  |  |  |  |  |  |  |  |  |  |  |
| Paper 30 et al |  |  |  |  |  |  |  |  |  |  |  |  |
| Paper 31 et al |  |  |  |  |  |  |  |  |  |  |  |  |
| Paper 32 et al |  |  |  |  |  |  |  |  |  |  |  |  |

EMS: Emergency medical service; Y: yes; N: no; ?: unclear/not applicable
